# Empowering vital fruit crops with enhanced nutritional contents

**DOI:** 10.3389/fpls.2025.1519673

**Published:** 2025-02-24

**Authors:** Avinash Jha, D. K. Jayswal, Deep Shikha, Adyant Kumar, Feza Ahmad

**Affiliations:** ^1^ Department of Plant Breeding & Genetics, Veer Kunwar Singh College of Agriculture, Bihar Agricultural University, Dumraon, Buxar, India; ^2^ Department of Horticulture (Fruit & Fruit Technology), Bihar Agricultural College, Sabour, Bihar Agricultural University, Sabour, Bhagalpur, India; ^3^ Department of Agronomy, Dr. Kalam Agricultural College, Bihar Agricultural University, Arrabari, Kishanganj, India

**Keywords:** biofortification, underdeveloped nations, gross domestic product, climate change, malnutrition, hidden hunger, conventional etc

## Abstract

Increasing the nutritional value of any crop plant through various Conventional or non-Conventional methods is known as Biofortification. Deficiency of proteins, essential amino acids, vitamins and minerals leads to ailing health and increased vulnerability to various diseases, which in turn lead to uncountable and unpredicted loss in Gross Domestic Product leading to poor economic growth of the country. It is forthcoming and cost-effective approach that will provide a balance of micronutrient deficiency among the people of developing & underdeveloped nations not having the availability to diverse nutritional access. The Biofortified varieties not only provide required calories but also essential nutrients needed for proper growth and development of an individual. It is advantageous in combating malnutrition and hidden hunger by enhancing the micronutrient content of commonly consumed fruits. By increasing essential vitamins, minerals, and beneficial compounds through methods like traditional breeding, genetic engineering, and agronomic practices, biofortified fruits provide a sustainable solution to address deficiencies in regions with limited access to diverse foods. For instance, mango, guava, papaya, and citrus have been improved to offer higher levels of nutrients such as iron, zinc, vitamin C, and beta-carotene. This makes biofortified fruits a cost-effective way to enhance nutrition, particularly for vulnerable populations, helping to reduce the risks associated with hidden hunger and malnutrition. One of the important targets of United Nation is to provide fortified food enriched with important minerals to the targeted undernourished population in different parts of the world. The lack of essential nutrients, notably minerals such as iron (Fe), zinc (Zn), and vitamin A, is one of the main causes of “hidden hunger”, especially in underdeveloped nations. The review covers most of the important aspects of Biofortification in important fruit crops.

## 1 Introduction

In the present scenario food security is major concern for most of the countries due to non-predictive Climate Change and increasing Population. Fortification of various crop plants in present and future scenario will be a major player in overcoming global hunger. According to United Nation, malnutrition & hidden hunger is severely affecting the people of under developed and developing nations like Africa and Southern Asia. Out of the total Infants deaths, maximum is attributed by these two factors only. Some of the Developed countries like Canada, USA & Europe also face deficiencies of Iron, Zinc, Iodine, and Vitamin A & D. Micronutrient malnutrition affects over 2 billion people around the world and causes serious health problems due to a lack of important vitamins and minerals like iodine, iron, zinc, and vitamin A. These nutrients are needed in small amounts for the body to function properly, but poor diet and problems with absorption, along with factors like food insecurity and poor healthcare, lead to deficiencies, especially in pregnant women and children. Iron deficiency, which causes anemia, affects more than 2 billion people (Benoist et al., 2008), while zinc deficiency affects about 1.1 billion people, leading to weakened immunity and growth problems (Frassinetti et al., 2006). Solving micronutrient deficiencies is important for improving health, especially in developing countries.

One of the major goals of World Health Organization (WHO) and Consultative Group on International Agricultural Research (CGIAR) is focusing on development of high yielding Biofortified crops. Considering the global scenario more than two billion people suffer micronutrient deficiency, 820 million people are undernourished, out of which 149 million children’s below 5 years of age are affected, and the condition is more serious in South Asian Countries. Coming on to the Indian scenario where 21.9% of population lives in extreme poverty hidden hunger and malnutrition is becoming serious day by day. In India 15.2% of people are undernourished, 38.4% of the children less than five years of age are stunted, 21.0% are wasted and 35.7% of the children are under-weight, 58.4% of the children below 5 years, 53% of the adult women and 22.7% of adult men are affected due to anemia. In countries like India a loss of 12 billion dollars is estimated every year due to these deficiencies in direct or indirect forms (Yadava et al., 2020). The high global consumption of bananas and mangoes highlights their significant potential for biofortification, which can play a crucial role in addressing micronutrient deficiencies. In Uganda, where banana consumption is exceptionally high, biofortifying bananas with essential nutrients like vitamin A, iron, and zinc could greatly enhance public health by improving nutrition in populations heavily reliant on bananas. Similarly, with mangoes being the second most consumed tropical fruit globally, biofortification can increase their nutritional value, helping to combat common deficiencies in regions where they are a dietary staple. As countries with high fruit consumption, such as India, the U.S., and Dominica, continue to rely on bananas and mangoes, biofortifying these widely consumed fruits offers an efficient, sustainable approach to combating malnutrition and improving overall health (FAO, 2020; World Population Review, 2023; Food and Agriculture Organization, 2023).

Malnutrition, particularly in developing countries, is not only driven by a lack of macronutrients—carbohydrates, proteins, and fats—but also by micronutrient deficiencies. While macronutrients are essential for providing energy and supporting body functions, micronutrient deficiencies, despite an adequate food supply, can lead to serious health problems. People in these regions often rely on staple foods like cereals and beans, which provide sufficient macronutrients but are low in essential vitamins and minerals. This imbalance exacerbates malnutrition, especially for vulnerable groups like children and pregnant women. Biofortification, which enhances the nutrient content of staple crops, can help improve the micronutrient intake without altering the availability of macronutrients. This strategy can address hidden hunger by improving the overall nutritional quality of the diet and reducing reliance on expensive or diverse foods (Kiran et al., 2022).

Nutrient movement and distribution within fruit trees are key to their growth, fruit production, and quality. Macronutrients like nitrogen (N), phosphorus (P), potassium (K), and calcium (Ca) are essential for these processes. While most macronutrients can move throughout the plant, calcium is less mobile, and its movement depends on water flow and areas of the plant with higher transpiration. Research has shown that calcium moves mainly through the xylem, and its distribution is influenced by the plant’s need and water flow. Nutrients from older leaves, pruning, and decomposing plant material also help maintain nutrient levels. Effective management of these nutrients, especially calcium, is important for fruit quality, as deficiencies can affect development. Ongoing studies focus on improving how nutrients are absorbed and distributed to boost tree health and fruit yield. New technologies to track nutrient flow and better nutrient management strategies aim to make fruit production more efficient and environmentally friendly (Kalcsits et al., 2020).

The work has been practiced in most of crops but this article mainly deals with Biofortification of the horticultural crops like Pomegranate, Banana, Mango, Grape, Papaya, Apple, Pear etc.

## 2 Biofortification strategies

**Figure 1:**
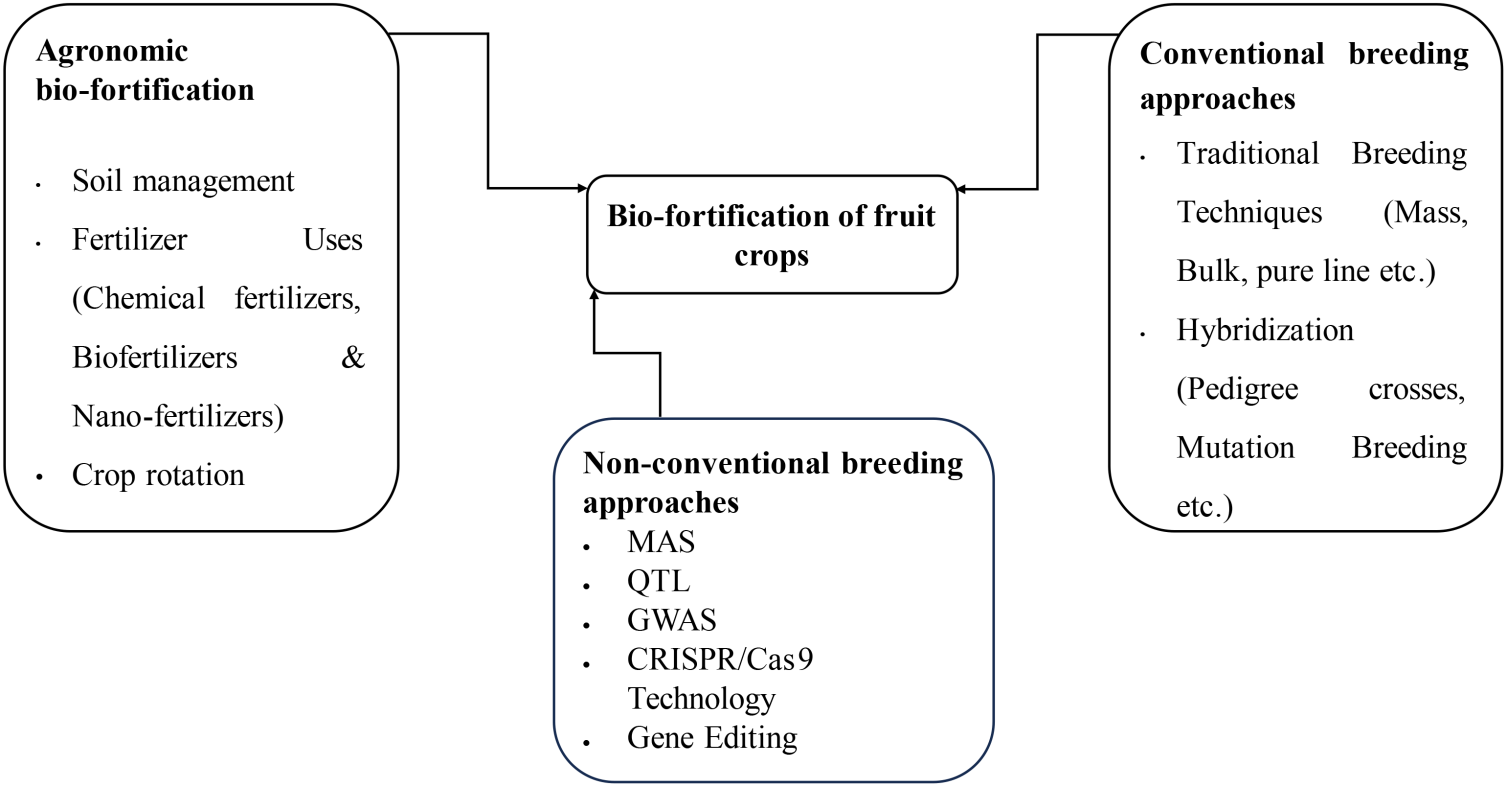
Methods of bio-fortification of fruit crops.

**Figure 2:**
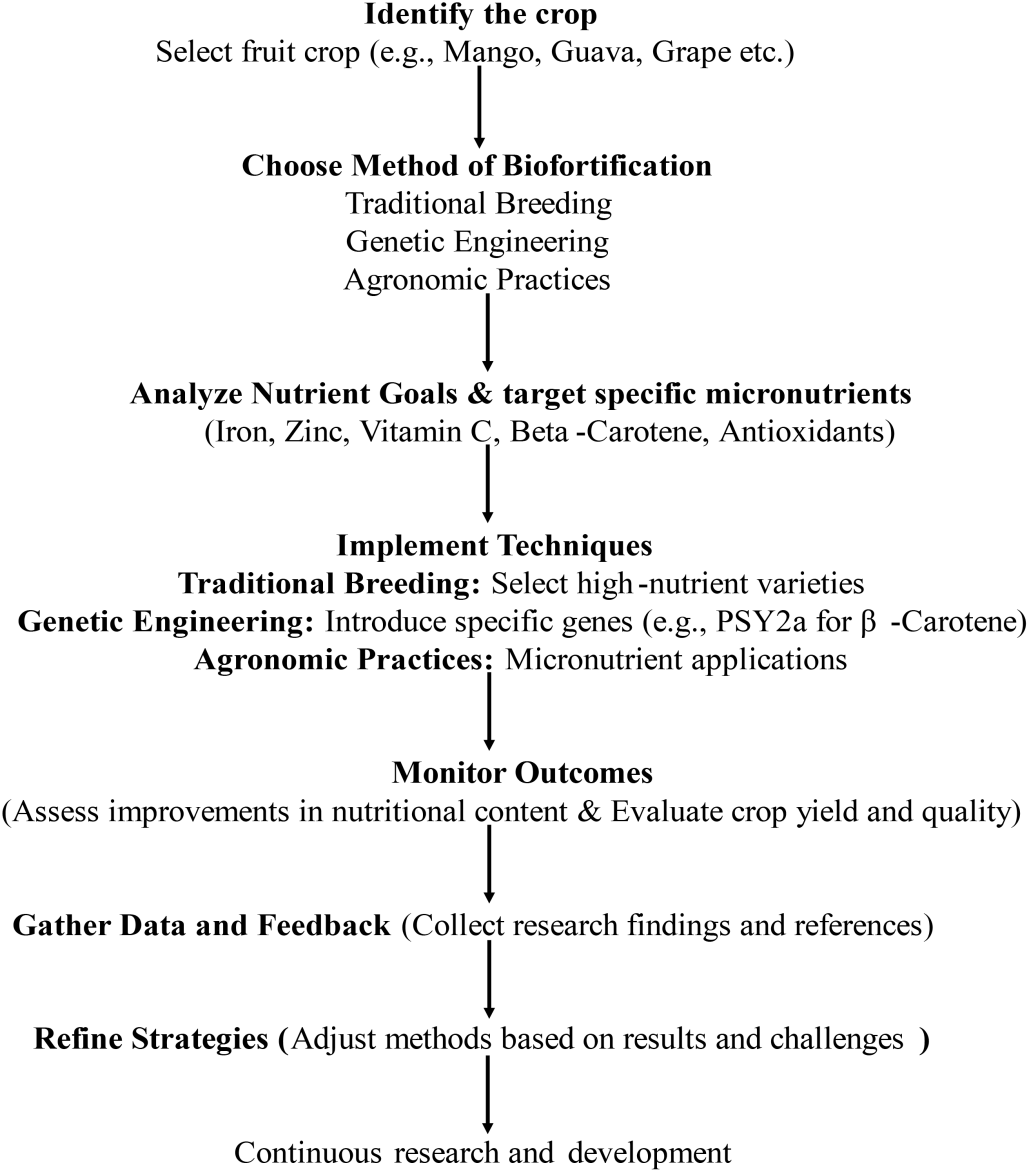
Procedure for bio-fortification of fruit crops.

The various Biofortification strategies as presented in the **Figure 1** include Agronomic, Conventional Breeding approaches and non-conventional breeding approaches like Genetic Engineering. Biofortification of fruit crops can be done through the procedure as explained in the **Figure 2**. All the strategies have been explained

### 2.1 Agronomic biofortification

Increasing the nutrient content in commercially adapted varieties through various agronomic means like fertigation, foliar application or through normal fertilizer application without affecting the threshold limit of other nutrients is known as Agronomic Biofortification. It is the fast safe and economical effective way of Biofortification with minimum efforts to enrich crops along with public acceptance. Most of the present-day experiments are based on this practice however, commercial exploitation has certain limitations like Low nutrient use efficiency (1-5%) for micronutrients like iron, zinc, copper, etc. which limit the uptake of applied micronutrients by plants. However, using heavy dosages of these micronutrients over a long period of time can lead to toxicity as most of the micronutrients remain in soil for longer duration of time. Some of the uncontrolled factors like changing weather conditions and Geographic variation in soil micronutrient deficiencies limit its usage up to some extent.

Agronomic biofortification focuses on improving the nutritional content of crops through farming practices. For example, pomegranate has been enhanced through agronomic techniques to increase iron, zinc, and vitamin C levels (Yadava et al., 2020). Similarly, grapes have been improved through agronomy to boost polyphenols, anthocyanins, resveratrol, and vitamins C and K (Di Lorenzo et al., 2019). Watermelon has also been biofortified using agronomic practices to increase lycopene, protein, and amino acids (Perkins et al., 2001). Additionally, crops like citrus, pear, and strawberry have been improved through agronomic methods as explained in **Table 1** to increase vitamins, minerals, and antioxidants (Salonia et al., 2020; Zhang et al., 2021; Singh et al., 2022). These techniques are essential in enhancing the nutritional quality of fruit crops.

**Table 1:** Nutritional fortification of important fruit crops.

S.No.	Fruits	Fortified with micronutrient	Referances
1	Pear	Foliar spray of 1.5% ZnEDTA & Foliar application of KI and KIO_3_ (0.5, 1.0 and 2.5%)	Liu et al., 2023; Budke et al., 2021; Mengjiao et al., 2023
2	Apple	Foliar application of KI and KIO_3_ (0.5, 1.0 and 2.5%)	Budke et al., 2021
3	Banana	Injection of ZnSO_4_ 7 H_2_O in pseudostem @ 1, 2 and 4%	Guevara et al., 2021
4	Grapes	Spraying Zn & ZnSO_4_ @ (150, 450, and 900 g ha−1)	Daccak et al., 2022
5	Strawberry	900 ppm cycocel	Kumar et al., 2012
6	Mango	ZnSO_4_ and FeSO_4_ (0.5% each)	Mahida et al., 2023
7	Pomegranate	2.6 mM FeSO_4_ (0.26%)	Davarpanah et al., 2020
8	Citrus	22.35 mg Zinc (Foliar spray)	Bhantana et al., 2022

#### 2.1.1 Nutrient transport and biofortification in plants

Plants primarily absorb nutrients through their roots, which transport them via the xylem. Understanding the availability of micronutrients in soil and their uptake by plants is crucial for enhancing plant nutrition, as confirmed by Bhardwaj et al. (2022). Different plant types employ distinct strategies for nutrient absorption. For instance, non-grassy plants (dicots) enhance iron (Fe) solubility in low-iron conditions, while grassy plants (monocots) utilize compounds called phytosiderophores to capture iron from the soil (Hell and Stephan, 2003).

Various transport proteins, such as those in the ZIP family, facilitate the uptake of key nutrients like zinc (Zn) and copper (Cu) (Eckhardt et al., 2001). When nutrients flow into the shoots, they promote root absorption and help regulate nutrient levels within the plant. After entering the xylem, nutrients are pulled up to the leaves, where they first act as sinks (nutrient importers) and later as sources (nutrient exporters) once photosynthesis exceeds the leaves’ energy needs (White and Broadley, 2009).

Nutrient movement differs within the plant: roots send nutrients to leaves via the xylem, while leaves transport them to fruits and seeds through the phloem. Some nutrients, like selenium (Se) and magnesium (Mg), move quickly through the phloem, whereas others, like iron and zinc, exhibit limited mobility. This limitation makes fruits and grains less nutrient-rich compared to leafy vegetables (White and Broadley, 2003). Foliar applications-spraying nutrients on leaves-can efficiently deliver micronutrients directly to the phloem, making this method valuable for biofortification.

Grains receive nutrients through a single vascular bundle, primarily storing iron and zinc in the embryo and aleurone layer. Specific proteins aid in transporting and storing these nutrients (Zhang et al., 2007). Enhancing beneficial substances, such as vitamins, while reducing harmful compounds called antinutrients (like phytates) can significantly improve nutrient absorption in the human diet. These strategies are supported by the findings of Bhardwaj et al. (2022) and can greatly assist in biofortification efforts, making crops more nutritious (Sancenon et al., 2003).

#### 2.1.2 Biofertilizers: enhancing plant growth

Biofertilizers are microbial products made from beneficial microorganisms that boost the growth and productivity of plants (Sahoo et al., 2013). These organisms are known as plant growth-promoting microorganisms and are valuable because they are inexpensive, easy to produce, and environmentally friendly. They enhance the supply and availability of nutrients in the soil, leading to better nutrient content in plants (Sahoo et al., 2013).

For instance, zinc (Zn) biofortification in crops like corn is aided by cyanobacteria such as *Azotobacter* and *Anabaena*, as well as the bacterium *Bacillus aryabhattai* (Prasanna et al., 2015). Similar benefits have been observed in wheat and soybeans (Ramesh et al., 2014). Using microbial interventions is recommended to address zinc deficiencies in soils (Dotaniya et al., 2016).

Arbuscular mycorrhizal fungi, like *Rhizophagus irregularis*, improve root development and help plants absorb essential nutrients like phosphorus (P), nitrogen (N), zinc (Zn), copper (Cu), manganese (Mn), and iron (Fe). This fungus has been shown to increase minerals in medicinal plants such as *Mentha pulegium* and *Petroselinum hortense* (Gashgari et al., 2020). Additionally, species of *Pseudomonas*, including *Pseudomonas chlororaphis*, enhance iron uptake and overall plant health (Sharma and Johri, 2003).

However, there are challenges in using biofertilizers. Identifying the right microorganisms for specific crops can be difficult, and biofertilizers may have a short shelf life due to variations in different environments. Improper storage and application can also hinder the benefits of biofortification.

Given these advantages, biofertilizers can be effectively applied to fruit crops such as strawberries and citrus fruits. For instance, using *Rhizophagus irregularis* can enhance root development and nutrient uptake, leading to better yields and nutrient-rich fruit in strawberries (Flores-Felix et al., 2015). Additionally, applying *Pseudomonas chlororaphis* could improve iron absorption in citrus trees, boosting fruit quality. Similarly, beneficial cyanobacteria can help address zinc deficiencies in strawberries, ensuring healthier plants and more flavorful berries. By leveraging these microbial interventions, fruit growers can improve growth and productivity while facing challenges posed by climate change and soil nutrient deficiencies.

#### 2.1.3 Nano-fertilizers: a new approach to nutrient delivery

Nano-fertilizers are fertilizers where the active ingredients are reduced to tiny particles, typically ranging from 1 to 100 nanometers. This size allows them to be effectively dispersed or encapsulated in host materials (Bhardwaj et al., 2022). Recently, nano biofortification has gained traction as a successful method to improve nutrition in crops like wheat (Khan et al., 2021). These tailored fertilizers have the potential to revolutionize agriculture by offering safe, targeted, and easy-to-apply nutrient delivery systems (Dimkpa and Bindraban, 2016; Manjunatha et al., 2016; Rai et al., 2018).

The high surface area of nano-fertilizers makes them more efficient and allows for slow-release nutrient supply to crops. They can include various nanoparticles, such as zinc oxide, silica, iron, and titanium dioxide, as well as core-shell quantum dots containing essential nutrients like Zn, Fe, Mn, and Cu (Prasad et al., 2015; Bhardwaj et al., 2022). The effectiveness of these nano-fertilizers depends on factors such as the plant species, size, concentration, and composition of the nanomaterials (Thakur et al., 2018). They are specifically engineered to address nutrient deficiencies, ensuring that plants not only grow but also accumulate essential nutrients in their edible parts (Li et al., 2016).

For example, a study by Dapkekar et al. (2018) showed that durum wheat treated with zinc complexed chitosan nanoparticles (Zn-CNP) increased grain zinc content significantly, even at lower concentrations compared to traditional zinc sulfate. Similarly, Hussain et al. (2021) found that foliar application of zinc oxide nanofertilizer boosted zinc levels in wheat plants. The use of chitosan-complexed zinc nano-fertilizers also led to higher grain zinc content and specific nutrient accumulation in seeds (Doolette et al., 2020).

Nanotechnology can be effectively applied to fruit crops to enhance their nutritional quality. By utilizing tiny particles known as nanoparticles, we can improve the ability of fruit plants to absorb essential nutrients. These nanoparticles can be applied to the seeds or sprayed on the leaves, facilitating better nutrient uptake.

For example, nanoparticles can induce a movement called “Brownian Motion,” which helps distribute nutrients more efficiently within the fruit plants. The smaller the particles, the more effectively the plants can absorb nutrients through their roots. This approach has shown promising results in increasing the nutrient levels in fruit crops, making them healthier for consumption (Jiang et al., 2018; Torre-Roche et al., 2020).

Further studies have demonstrated that using nano-iron and nano-manganese fertilizers can improve nutrient uptake and plant growth. For instance, applying nano-Fe2O3 stimulated root growth better than conventional iron forms, while nano-Mn showed increased manganese accumulation in seeds and enhanced overall plant growth (Pradhan et al., 2013; Dimkpa et al., 2018). These advancements in nano fertilizers can also be effectively applied to fruit crops. For example, using zinc and iron nano fertilizers can enhance nutrient uptake in fruit trees like apple and citrus, leading to improved fruit quality and yield. The slow-release properties of these fertilizers can ensure that fruit crops receive consistent nutrition throughout their growing season, ultimately increasing their resistance to stress and enhancing flavor and nutritional content. By adopting nano-fertilizers, fruit growers can address specific nutrient deficiencies while promoting healthier, more productive crops.

### 2.2 Conventional breeding approaches

Breeding interference had played a significant role in Biofortification of important crops, where several traits are identified based on multiple environment stability assessment Models affecting the fortification pathway. Various breeding approaches like exploring variability present in natural population via Mass, Bulk and pure line selection or creation of variability through pedigree crosses or through induced, spontaneous or natural mutations in a population by targeted exploitation of mechanism favoring Biofortification, as it has been used to create grape varieties with more nutrients. By selecting specific plants, breeders aim to increase the levels of important compounds like polyphenols and anthocyanins in grape berries. These compounds help to improve the antioxidant properties and health benefits of grapes (Di Lorenzo et al., 2019). The transfer of required gene favoring Biofortification pathways through Backcross breeding will serve as an important tool in present and future times to enhance the existing varieties and lines of commercially important hybrids with increased nutrient content without hampering its original yield. The use of wild relatives of crop plant for transferring required gene favoring Biofortification is yet to be used at larger scale as it is a time-consuming process and due to linkage drag of unwanted genes associated with wild species. Sometimes desirable genes of interest rarely express due to gene interactions and other uncontrolled factors.

Various fruit varieties have been developed through conventional breeding to improve their nutritional value. In Uganda, HarvestPlus developed banana varieties like Apantu, Bira, Pelipita, Lai, and To’o (**Table 2**), which are rich in Vitamin A. In India, IARI developed several mango varieties such as Amarpali, Pusa Arunima, Pusa Surya, Pusa Pratibha, Pusa Peetamber, Pusa Lalima, and Pusa Shreshth, all high in beta-carotene and Vitamin C (**Table 2**). The Ataulfo mango, known for its beta-carotene and Vitamin C content, was developed by the USDA Agricultural Research Service in Mexico. Additionally, IARI in India developed the Pusa Navrang grape, which is packed with antioxidants.

**Table 2:** Biofortified Varieties developed in important fruit crops.

S.No.	Crop	Variety	Trait	Country	References
1	Pomegranate	Solapur Lal	Iron & vitamin C	India	National Research Centre on Pomegranate in Pune
2	Banana	Apantu, Bira, Pelipita, Lai, To’o	Vitamin A	Uganda	HarvestPlus. Biofortified crops released (2022)
3	Mango	Amarpali, Pusa Arunima, Pusa Surya, Pusa Pratibha, Pusa Peetamber, Pusa Lalima, Pusa Shreshth	Beta-carotene, Vitamin C	India	IARI, India
		Ataulfo	Beta-carotene, Vitamin C	Mexico	USDA Agricultural Research Service
4	Grapes	Pusa Navrang	Antioxidants	India	IARI, India
5	Aonla	NA-7	Vitamin C	India	Iqbal et al., 2015

Avnee et al. (2023) highlighted various crops and the specific genes involved in enhancing their micronutrient content. For instance, banana utilizes the PSY2a gene to increase β-Carotene levels, while apple employs stilbene synthase for antioxidant production (Flachowsky et al., 2010). Sweet potato benefits from genes like IbMYB1 and npt II to boost anthocyanin, carotenoids, and antioxidants, alongside Crtl, CrtB, CrtY, and LCYe for β-Carotene (Kim et al., 2013; Park et al., 2015). In cassava, multiple genes including Erwinia crtB and phytoene-synthase contribute to β-Carotene enhancement, while the EFA1 gene and Ferritin FEA1 are linked to iron content (Sayre et al., 2011; Telengech et al., 2015; Sharma et al., 2017).

### 2.3 Dietary diversification and supplementation

To combat micronutrient malnutrition sustainably, dietary diversification is key. This approach encourages consuming a wide variety of healthy foods, including fruits, vegetables, and different grains. Success depends on factors like affordability, dietary habits, accessibility, and bioavailability of nutrients. Raising awareness about nutrition through media can help promote diverse diets.

Enhancing dietary diversity can be achieved by improving agricultural production, offering a greater variety of foods, and promoting better food processing and preparation methods. Additionally, combining dietary diversification with supplementation (like tablets) and fortification can effectively address specific nutrient deficiencies in the population (Lee et al., 2011).

### 2.4 Microbes mediated biofortification of fruit crops

Microbial biofortification is a useful way to improve the nutritional quality of crops by using helpful microorganisms like Plant Growth-Promoting Rhizobacteria (PGPR) and Arbuscular Mycorrhizal Fungi (AMF). PGPR can boost the yield and nutrient content of crops, especially legumes, by helping them fix nitrogen, which is important for their growth. AMF work with plant roots to help them absorb essential nutrients like phosphorus and micronutrients such as selenium. For example, garlic treated with Glomus intraradices has shown a tenfold increase in selenium content, showing how effective these microbes can be in improving nutrition (Roriz et al., 2020; Larsen et al., 2006).

Additionally, specific types of microbes have been successfully used on different crops to increase their micronutrient levels. For instance, using zinc-solubilizing bacteria has raised the zinc content in tomatoes from 2.06 mg to 2.87 mg per 100 g. This method not only makes crops healthier but also reduces the need for chemical fertilizers, which is better for the environment. However, there are challenges, such as not knowing enough about the micronutrient levels in seeds and how climate change affects nutrient levels, which make it hard to widely use microbial biofortification (Thakur et al., 2022).

This approach can also be applied to many fruit crops, helping to improve their nutritional quality and address vitamin and mineral deficiencies in people’s diets around the world.

### 2.5 Biofortification of fruit crops through biotechnological tools

Marker-assisted breeding (MAB) is an effective method used to improve the nutritional quality of fruit crops. This technique uses molecular markers to identify and select specific traits that enhance nutrient content in plants. By using MAB, breeders can quickly add desirable traits to fruit varieties, ensuring they are not only high-yielding but also rich in essential vitamins and minerals.

Biotechnology tools, such as genetic engineering and genome editing, work well with marker-assisted breeding. These tools allow for precise changes in the plant’s genetic makeup, enabling the introduction of specific genes that boost the availability of important nutrients. For example, transgenic methods can be used to add genes that increase the levels of vital nutrients like iron and zinc in the edible parts of fruit crops (Bouis et al., 2017; Athar et al., 2020).

The CRISPR/Cas9 genome editing technology is another innovative method for improving the nutritional quality of fruit crops. This advanced technique allows for targeted changes in the plant’s DNA, making it easier to enhance specific traits related to nutrient content. By combining marker-assisted breeding with these biotechnological tools, researchers can develop fruit varieties that are not only more nutritious but also better able to withstand environmental challenges as presented in **Table 3**.

**Table 3:** Details of the fortified crops with the help of Genetic Engineering are as follows.

Crop	Variety/Genes Involved	Micronutrient	References
Banana	PSY2a	β-Carotene	Waltz, 2014
Apple	Stilbene synthase	Antioxidant	Flachowsky et al., 2010
Sweet Potato	IbMYB1, npt II	Anthocyanin, carotenoids, antioxidants	
			Kim et al., 2013
	Crtl, CrtB, CrtY, LCYe	β-Carotene	
			Park et al., 2015
Cassava	Erwinia crtB, phytoene-synthase gene, & Arabidopsis 1-deoxyxylulose-5-phosphate synthase	β-Carotene	
			Sayre et al., 2011 & Telengech et al., 2015
	EFA1 gene	Fe	
			Sharma et al., 2017
	Ferritin FEA1	Fe	
			Sayre et al., 2011
	ASP 1, Zeolin Protein	–	
			Sayre et al., 2011

RNA interference (RNAi) technology has been used to create cassava plants that are resistant to cassava mosaic disease (CMD) and cassava brown streak disease (CBSD). This technology works by introducing small RNA molecules that target and break down the viral RNA, stopping the virus from spreading in the plant. Field trials have shown that these genetically modified cassava plants are resistant to both diseases and grow normally, even in conditions where the diseases would usually cause major damage. This innovation shows the potential of RNAi in controlling viral diseases in important crops, helping to ensure food security in areas where cassava is a key crop (Jain, 2024).

### 2.6 Advancements in biofortification of fruit crops using new plant-breeding techniques:

According to Bharti and Chimata (2019), New Plant-Breeding Techniques (NBTs) such as CRISPR and ZFN offer promising solutions for biofortifying fruit crops by improving their nutritional content more efficiently. These techniques allow for the development of fruit varieties that are richer in essential vitamins and minerals like vitamin C, iron, and zinc. Besides enhancing nutrient levels, NBTs can help address challenges such as pest resistance and environmental stresses, making fruit production more sustainable. By reducing the need for pesticides and optimizing resource use, these techniques contribute to the creation of nutrient-dense fruits that benefit farmers, consumers, and the environment. According to the study, Site-Directed Nucleases (SDN) and Oligonucleotide-Directed Mutagenesis (ODM) are two methods under NBTs that can be used to precisely modify genes in fruit crops, improving nutrient uptake and enhancing health benefits without introducing foreign DNA. These techniques offer non-GMO solutions for biofortifying fruits, making them an acceptable option for public concerns.

Bharti and Chimata (2019), also found that Cisgenesis and Intragenesis are other innovative techniques that can be used for transferring beneficial genes within the same or closely related species. These approaches allow for the introduction of traits like disease resistance and higher nutrient content without the need for foreign DNA, making biofortified fruits similar to conventionally bred ones. Additionally, RNA-Dependent DNA Methylation (RdDM) is a technique that can regulate gene expression to enhance nutrient production, improving vitamin and antioxidant content in fruits. Since RdDM modifies gene expression rather than the genetic sequence, it provides a way to biofortify fruits without foreign DNA. Techniques like grafting, combining genetically improved rootstocks with non-GM scions, can further enhance fruit yield and nutritional quality. According to the study, agro-infiltration, a method to test biofortification traits in fruit crops, offers a way to evaluate the effects of genetic modifications quickly, allowing for more effective breeding programs. Overall, these techniques can be used in the future to transform fruit crops, making them more nutritious, sustainable, and beneficial in addressing global malnutrition challenges.

### 2.7 Advancements and challenges in non-transgenic biofortification

Exploring the Role of Organic Fertilizers, Biofertilizers, and Nutri-Priming in Micronutrient Enhancement of Crops”: Biofortification through non-transgenic methods (Marques et al., 2021). offers several benefits for increasing micronutrient content in crops, utilizing approaches such as organic fertilizers, biofertilizers, and nutri-priming. Organic fertilizers, like manure and compost, are eco-friendly alternatives to inorganic fertilizers and have been shown to boost micronutrient levels in crops such as barley, wheat, and rice. However, they come with limitations, including the potential presence of harmful pathogens, antibiotics, and heavy metals, and the highly variable nutrient content, which makes precise nutrient delivery challenging. Similarly, biofertilizers, which use plant growth-promoting microorganisms, can enhance nutrient availability and crop growth, but their effectiveness is limited by factors like soil conditions, environmental factors, and the challenge of selecting the right microorganisms for specific crops. Despite the promise of biofertilizers, they often fail to fully replace traditional fertilizers in agroecosystems. Nutri-priming, another approach where seeds are soaked in nutrient solutions before planting, can increase micronutrient content, such as zinc in wheat and chickpeas. This method is low-cost and farmer-friendly but is also influenced by crop type, genotype, and environmental conditions, which can limit its effectiveness. Overall, while these non-transgenic biofortification methods have potential, their limitations, such as nutrient availability, environmental dependency, and lack of consistency, must be addressed to optimize their use in improving micronutrient intake, particularly in resource-limited settings.

### 2.8 Non-conventional approaches

The use of biotechnological tools like marker-assisted selection (MAS) and quantitative trait locus (QTL) maps by breeders or biotechnologists to identify gene of interest in large populations for use in breeding program is also one of the important aspects. Marker aided selection (MAS) leads to indirect selection of desired nutrient pathway favoring selection for Biofortification based on banding pattern of linked molecular markers. Various natural or created populations like Recombinant inbred lines (RILs), near isogenic lines (NILs) of interest are used for it. By the use of Genetic engineering technique or transgenic Breeding we can transfer gene of interest after cloning it across Genera and Species. Various Biological methods like Agro bacterium mediated gene transfer and non-biological methods like gene gun method, liposome mediated gene transfer, Micro injection method are yet to be used at larger scales for proper gene deployment favoring nutrient enhancement in required crop plants. For map-based cloning of genes of interest special mapping strategies can be used for rapidly targeting specific regions of the genome (Yu et al., 2021).

Various Molecular methodologies will serve as an important tool in future for development of Biofortified varieties by evaluation of genetic resources at molecular level and providing the clear-cut ideas of beneficial genetic resources for usage in further breeding programme (Shah et al., 2023). The use of CRISPR-Cas and Genome wide association mapping (GWAS) are the most advanced tool for success under present scenario. The CRISPR-Cas technology enables crop genome editing for qualitative enhancement in aroma, shelf life, oleic acid, anthocyanin, phytic acid, gluten etc. contents in several crop plants (Liu et al., 2021). Various genome edited golden crops like rice cultivar Kitaake has been developed by Knock-in, a 5.2-kb carotenogenesis cassette consisting of CrtI and maize PSY genes. The variety contains 7.9 μg/g dry weight (DW) β-carotene in the endosperm (Dong et al., 2020).

The use of new tools like Genome wide association mapping (GWAS) has opened the new door for fortification in important crops adapted to specific regions. GWAS can identify the loci associated with observed variations in phenotype for various agronomic traits among the Germplasm having nutrient deficit, which plays important role in regulation of the traits for using it as a genetic tool for pre breeding activities. Genome-wide association studies (GWAS) use high-throughput genomic technologies to scan entire genomes of large numbers of targets quickly, in order to find genetic variants correlated with a trait of interest. Accelerating breeding efforts for developing Biofortified bread wheat varieties necessitates understanding the genetic control of grain zinc concentration (GZnC) and grain iron concentration (GFeC) (Juliana et al., 2022). Scientists are trying to explore similar technique in most of the commercially important fruit crop.

## 3 The details of various Biofortified fruit crops are presented below

### 3.1 Pomegranate (*Punica granatum*)

The Pomegranate Solapur Lal is a high-nutrient hybrid (Bhagawa x [(Ganesh x Nana) x Daru]) variety developed by the ICAR-National Research Centre on Pomegranate in Pune (**Table 2**). Released in 2017, it is designed for semi-arid regions and yields 23 to 27 tons of fruit per hectare. It contains significantly higher levels of iron (5.6–6.1 mg/100g), zinc (0.64–0.69 mg/100g), and vitamin C (19.4–19.8 mg/100g) compared to the Ganesh and Bhagwa varieties, which have lower nutrient levels (Yadava et al., 2020). This variety matures 15-20 days earlier than Bhagwa, with a growing period of 160-165 days and an average yield of 35-39 kg per plant. It also has higher amounts of vitamin C, anthocyanins, and zinc, though it has a harder seed coat than Bhagwa.

Davarpanah et al. (2020) found that early season foliar fertilization with 2.6 mM FeSO_4_ significantly increased pomegranate fruit yield and quality. The treatment led to a 20–31% increase in yield, improved the number of fruits per tree, and enhanced juice content, total soluble solids, and sugar levels. FeSO_4_ also lowered juice acidity and raised the maturity index, though it slightly reduced antioxidant activity and phenolic compounds in the juice. This study highlights FeSO_4_ as an effective method for boosting pomegranate yield and fruit quality in high pH soils.

### 3.2 Banana (*Musa acuminata*)

Efforts to develop biofortified bananas focus on improving their nutritional content through traditional breeding and genetic engineering. Traditional breeding aims to increase levels of provitamin A carotenoids, like beta-carotene, to help combat vitamin A deficiency, especially in regions where bananas are a staple food (Davey et al., 2009). Genetic engineering has also been used to enhance bananas’ nutritional value. For example, the Golden Banana has been developed to have higher levels of provitamin A carotenoids to address vitamin A deficiency (Arango et al., 2010). Additionally, researchers are working on increasing other nutrients like iron and zinc in bananas (Naqvi et al., 2009). Efforts are also being made to reduce harmful compounds, such as acrylamide, which can form during cooking and may be a carcinogen (Joshi et al., 2017a). Red bananas, such as “Red Dacca” and “Red Cavendish,” offer additional health benefits compared to yellow bananas. They have higher vitamin C content, potassium, dietary fiber, and antioxidants like anthocyanins, which can provide antioxidant and anti-inflammatory benefits (Lobo et al., 2010). Researchers are also using RNA interference to combat viral infections in bananas. For instance, transgenic plants with silenced viral components have shown promise in eliminating the bunchy top virus (Shekhawat et al., 2012).

Breeding of bananas is challenging because most commercial varieties are sterile and cannot crossbreed easily. However, genetic engineering is effective for bananas since they are typically grown from plant parts, which reduces the risk of spreading modified genes. Genetically modified bananas can be grown alongside regular ones without affecting their diversity. Unfortified bananas have 0.4 mg of iron per 100 grams, while fortified bananas can have up to 2.6 mg. Efforts are being made to increase bananas’ vitamin A, vitamin E, and iron levels. Research at Queensland University of Technology (QUT) has developed bananas with higher levels of these nutrients and improved resistance to diseases like Banana Bunchy Top Virus (QUT 2024). Similar research is underway in India with varieties such as Grand Nain and Rasthali. The Donald Danforth Plant Science Center is also developing bananas with high provitamin A for Nigeria and Kenya, with a new variety expected to be available in future.

The Coordinated efforts of TNAU, Coimbatore and IIHR Bangalore along with Queensland University of Technology, Australia under banana Biofortification programme Grand Nain and Rasthali Varieties of Banana will be Fortified with (Provitamin A) in first phase and later these varieties will be incorporated with tolerance for Banana Bunchy Top virus and Fusarium Wilt disease, similar work has been done to increase provitamin A content to 20 ppm by Donald Danforth Plant Science Center for locally adapted varieties for release in Nigeria & Kenya (Prasad et al., 2015). The identification and diffusion of well adapted varieties with high Provitamin A content especially in endemic regions (Bouis and Saltzman, 2017) has been adopted for the dissemination of high Provitamin A content of cooking and dessert bananas in Burundi, Rwanda and DR Congo (Andersson et al., 2017 and Ekesa et al., 2017). Some researchers have tried to reduce acrylamide which is a carcinogenic toxic substance produced in banana while cooking without hampering the original nutritional quality (Joshi et al., 2017b).

### 3.3 Mango (*Mangifera indica*)

Biofortification of mangoes (Mangifera indica) aims to boost their nutritional value by increasing key micronutrients and phytochemicals. Although research on mango biofortification is limited, both traditional breeding and modern biotechnological methods have been explored. A study by Padmesh et al. (2013) examined how conventional breeding could improve the nutrient content of mangoes. The researchers found significant differences in mineral levels like iron, zinc, and calcium among various mango types, suggesting that breeding could produce mango varieties with higher mineral content. Genetic engineering is another approach being used to enhance mango nutrition. Saranya et al. (2017) worked on genetically modifying mango plants to increase their beta-carotene, a precursor to vitamin A. They introduced a gene from another plant that helps produce carotenoids, resulting in mangoes with higher beta-carotene levels. Agronomic practices also affect mango nutrition. Dhotra et al. (2021) tested the effect of applying micronutrients such as calcium nitrate, boric acid, and zinc sulfate to mango trees before flowering. Their study found that these nutrients enhanced the growth, yield, and quality of Dashehari mango fruits.

Mahida et al. (2023) found that agronomic biofortification, particularly using foliar sprays of zinc and iron, is an effective method to boost nutrient levels in crops. Their 2016-2017 field study on mango cv. Kesar showed that applying 0.5% FeSO_4_ and 0.5% ZnSO_4_ increased the nutrient content in both pulp and peel. The highest concentrations of zinc and iron were observed with the combination of both micronutrients, offering a promising approach to enhance mango nutrition.

### 3.4 Guava (*Psidium guajava*)

Biofortification of guava aims to increase its nutritional value by boosting levels of essential nutrients and beneficial compounds. Traditional breeding methods have focused on developing guava varieties with higher concentrations of vitamin C, beta-carotene, iron, and zinc. These efforts help address micronutrient deficiencies and enhance the fruit’s overall nutritional profile (Navarro-Tarazaga et al., 2016). Additionally, genetic engineering techniques have been applied to further enhance guava’s nutritional content. By introducing specific genes into guava plants, researchers can increase the production of important bioactive compounds. For instance, the Paluma guava cultivar, known for its rich crimson pulp and high soluble solids content, has been developed to offer improved nutritional benefits and antioxidant properties. Alongside these genetic approaches, optimizing agricultural practices such as proper fertilization and irrigation can significantly boost nutrient uptake and accumulation in guava plants, contributing to effective biofortification (Sathya et al., 2019).

### 3.5 Grape (*Vitis vinifera*)

Grapes have been improved using both traditional breeding and modern genetic techniques to boost their health benefits. Traditional breeding focuses on increasing the levels of beneficial compounds like polyphenols and anthocyanins, which have antioxidant properties and are good for health (Di Lorenzo et al., 2019). This method involves selecting grape varieties that naturally have higher amounts of these health-promoting compounds. Modern genetic engineering also plays a role in enhancing grapes. This approach involves adding or modifying specific genes to increase the production of desirable compounds, such as resveratrol, which is known for its health benefits (Vezzulli et al., 2007). By making precise changes to the grape’s genetic makeup, scientists can improve the grapes’ nutritional value. Additionally, how grapes are grown—through different fertilization, irrigation, and canopy management techniques—can also impact their nutritional content (Cortell and Kennedy, 2006). Grapes are rich source of antioxidant with high levels of vitamin C and K. Pusa Navrang a new Cultivar of Grape introduced by Indian Agricultural Research Institute is rich in soluble solids like carbohydrates, organic acids, proteins, lipids, minerals and antioxidants (**Table 2**).

Zhao et al. (2020) conducted a study on biofortifying grapes with selenium (Se) and lithium (Li) using foliar application of organic fertilizers rich in these micronutrients. The study focused on five grape cultivars and evaluated the impact on vine vigour, fruit quality, and the levels of micronutrients and phenolic compounds. The results showed that the biofortification significantly increased the Se and Li content in the grapes, particularly in the skin, without adversely affecting vine vigour or fruit quality. However, the biofortification did influence the ions (mineral nutrients and trace elements) and phenolic compounds, with variations across cultivars. This method proved to be an effective strategy for enhancing the nutritional value of grapes, potentially improving their health benefits and increasing consumer awareness and consumption of these enriched fruits, especially in the Midwestern United States.

Daccak et al (2022) studied the biofortification of grapes with zinc (Zn) to help address micronutrient deficiencies in food. Zinc is important for human health, and the research focused on the Fernão Pires grape variety. The vines were sprayed with two types of zinc fertilizers, ZnO and ZnSO_4_, at three different concentrations (150, 450, and 900 g ha−1) during the growing season. The study looked at how zinc accumulated in the grapes, especially in the skin and seeds, and how it affected grape quality and winemaking. The results showed that the zinc levels in treated grapes were much higher than in the control grapes, with ZnO increasing zinc in the skin and ZnSO_4_ in the seeds. After winemaking, zinc content in the wine also increased by about 1.59 times with the 450 g ha−1 ZnSO_4_ treatment. While there were no significant changes in sugar, fatty acids, or color, the study concluded that biofortifying grapes with zinc is a good way to increase their nutritional value without harming their quality, making it a useful strategy for improving both grapes and wine.

### 3.6 Papaya (*Carica papaya*)

Biofortification of papaya focuses on improving its nutritional value through genetic engineering. One key approach is increasing provitamin A carotenoids, like beta-carotene, by introducing genes that enhance carotenoid production (Shankar et al., 2010). This helps address vitamin A deficiency in populations that rely heavily on papaya. Another focus is boosting essential minerals, such as iron and zinc, in papaya fruits using genetic engineering techniques (Maxwell et al., 2013).

Non-conventional tools like Genetic Engineering led the introduction of specific genes involved in carotenoid biosynthesis in locally adapted papaya Variety that has enhanced the beta-carotene content (Shankar et al., 2010). After collecting material from Centre for Tropical Agriculture, Southedge, QLD Australia (Devit et al., 2010), observed that the expression level of both lcy-β1 and lcy-β2 genes is similar and low in leaves, but lcy-β2 expression increases markedly in ripe fruit. Isolation of the lcy-β2 gene from papaya, that is preferentially expressed in fruit and is correlated with fruit colour, will facilitate marker-assisted breeding for fruit colour in papaya and should create possibilities for metabolic engineering of carotenoid production in papaya fruit to alter both colour and nutritional properties.

### 3.7 Apple (*Malus domestica*)

Researchers have identified apple varieties with increased levels of antioxidants such as phenolic compounds, flavonoids, and anthocyanins, which are associated with health benefits due to their antioxidant and anti-inflammatory properties (Strand et al., 2018). Proper nutrient management, like balanced fertilization, can improve the nutritional quality of apples by influencing their nutrient content (Tagliavini et al., 2019). Genetic engineering is being explored to enhance apples by introducing specific genes. For example, incorporating a stilbene synthase gene from grapevines can increase resveratrol, a potent antioxidant, in apples (Tian et al., 2017). This genetic modification aims to improve the antioxidant capacity of apples. Scientists have also modified apple plants to produce higher levels of anthocyanins and flavan-3-ols, beneficial compounds that contribute to the apple’s nutritional value (Flachowsky et al., 2010). Additionally, Arctic^®^ apples have been engineered to prevent browning by inhibiting polyphenol oxidases (PPOs), the enzymes responsible for browning. Arctic varieties include Fuji, Granny Smith, and Golden Delicious, with different types being released over the years (Lobato-Gomez et al., 2021).

### 3.8 Pear (*Pyrus communis*)

Biofortification in pears aims to boost their nutritional content and help with nutrient deficiencies. Although research on pears is less developed compared to other crops, there are some key strategies being explored. One method is conventional breeding, where pear varieties are selected and crossed to increase their levels of vitamins, minerals, and antioxidants. Another method is genetic modification, which involves adding or enhancing specific genes to improve nutrient levels. For example, scientists have studied boosting genes related to antioxidants like anthocyanins to enhance the antioxidant properties of pears (Han et al., 2016). Agronomic Biofortification in *Pyrus communis* L. variety Rocha, has been successfully deployed with Foliar application of 0.6 kg Ca (NO_3_)_2_ ha^−1^ or 1.6 kg CaCl_2_ ha^−1^, that promotes Ca Biofortification in leaves without any phytotoxity symptoms (Cardoso et al., 2018). Successful attempts for Zinc fortification were done by (Liu et al., 2023) with chelated and non-chelated Zinc, however it was observed that chelated ZnEDTA can be safely used at a higher spraying rate of 1.5% as compared to 0.1%–0.4% for non-chelated Zn sources. Pear is a major fruit crop spreaded worldwide, but its breeding is time taking process. To facilitate molecular breeding and gene identification (Zhang et al., 2021), have performed genome-wide association studies (GWAS) on eleven fruit traits. They identified 37 loci associated with eight fruit quality traits and five loci associated with three fruit phenological traits. Over the period of time new advantageous mutation eliminates variation in lined neutral sites indicating that traits like fruit stone cell content, organic acid and sugar contents might have been under continuous selection during breeding improvement. One candidate gene, *PbrSTONE*, identified in GWAS, has been functionally verified to be involved in the regulation of stone cell formation, which is one of the most important fruit quality traits in pear.

### 3.9 Aonla (*Phyllanthus emblica*)

It is well-regarded for its high vitamin C content and other healthful compounds. Traditional breeding methods focus on improving its nutritional profile through selection and cross-breeding techniques, aiming to enhance its beneficial properties further (Singh et al., 2022). In addition, genetic engineering offers the potential to introduce specific genes that could boost nutrient levels in aonla. However, this approach faces challenges, including regulatory hurdles and public concerns about genetically modified organisms (Chaurasia et al., 2009). Moreover, the use of black polythene mulch has been found to improve the quality of aonla fruits, particularly for the NA-7 variety (**Table 2**), by positively affecting various quality parameters (Iqbal et al., 2015).

### 3.10 Strawberry (*Fragaria × ananassa*)

Efforts to improve strawberries focus on making them more nutritious and beneficial for health (Singh et al., 2022). Genetic engineering has helped boost the vitamin C content and increase the plants’ resistance to diseases and environmental challenges (Borowski et al., 2016). For instance, scientists altered strawberries to have up to 47% less starch and 37% more soluble sugar by blocking a specific enzyme that converts sugar into starch (Park et al., 2006). Additionally, applying a 900 ppm cycocel spray to strawberries has been shown to enhance their quality by increasing their sweetness, vitamin C content, juice yield, and lowering their acidity (Kumar et al., 2012).

### 3.11 Citrus (*Citrus sinensis*)

To improve the nutritional content of citrus fruits, traditional breeding methods involve selecting and crossbreeding varieties that naturally have higher nutrient levels. For example, breeders focus on increasing the vitamin C content in citrus fruits (Salonia et al., 2020). Genetic engineering also plays a role in enhancing citrus nutrition. Researchers have introduced genes to boost iron levels in citrus by increasing iron transporters, which can help combat iron deficiency (Zhang et al., 2019). They have also worked on increasing pro-vitamin A carotenoids, like beta-carotene, and folate in citrus fruits (Pons et al., 2018). Additional techniques include using specific fertilizers and treatments to improve the fruit quality, such as applying nitrogen, potassium sulfate, and calcium chloride (Bakshi et al., 2017; Devi et al., 2018). These efforts aim to provide citrus fruits with better nutritional value, especially in areas with high vitamin A deficiency.

Bhantana et al. (2022) emphasized the importance of optimizing micronutrient concentrations, particularly zinc (Zn), for improving the mineral nutrition, yield, and quality of citrus fruits. The study found that the soil had a Zn concentration of 22.35 mg/Kg, which is considered marginally available for plant uptake. Before treatment, the top leaves of the citrus varieties Wenzhou and Nanfeng showed relatively low concentrations of essential nutrients like nitrogen (N), phosphorus (P), potassium (K), and Zn. The study concluded that biofortification with Zn (either through foliar or soil application) is effective when soil Zn levels are below the critical threshold of 20 mg/Kg. Micronutrient supplementation under these conditions can improve the yield and nutritional quality of citrus fruits.

### 3.12 Watermelon (*Citrullus lanatus*)

Lycopene is a carotenoid compound (Perkins et al., 2001) responsible for the red color in watermelon and is associated with various health benefits, including reducing the risk of certain cancers and cardiovascular diseases. Researchers have focused on increasing lycopene content through conventional breeding methods and genetic engineering techniques. Studies have shown success in developing watermelon varieties with higher levels of lycopene, contributing to enhanced nutritional value (Deng et al., 2023). highlights the potential of using nanoscale CuO particles to enhance disease management and nutritional quality of crops. Specifically, negatively charged nanospikes demonstrated significant benefits in both disease control and nutrient enhancement. Investigation from (Joshi et al., 2021) highlighted considerable natural variation in the concentrations of free amino acids and total protein across different watermelon accessions and regions, through a genome-wide association study (GWAS), they identified 188 SNPs linked to 167 candidate genes involved in seed-bound amino acids and total protein content. Analysis revealed that SNP clustering was independent of watermelon speciation and cultivar types, suggesting these genetic variations were not influenced by the domestication process. Significant candidate genes, such as Argininosuccinate synthase, were associated with 7% of the variation in arginine content, confirming their functional relevance. This study provides critical insights and a robust platform for future research aimed at developing watermelon varieties with superior nutritional attributes.

### 3.13 Blueberries (*Vaccinium corymbosum*)

In the study by (Mengist et al., 2020), a high-throughput *in vitro* gastrointestinal digestion model was used to assess the bioaccessibility of phenolic compounds in various cultivated highbush blueberry accessions. The results showed significant differences in the bioaccessibility of flavonoids and phenolic acids among different accessions and years, with some accessions exhibiting high relative and absolute bioaccessibility values. Acylated anthocyanins were found to have higher relative bioaccessibility compared to non-acylated anthocyanins. The study also suggested that both genetic and environmental factors influence phenolic bioaccessibility, with broad sense heritability estimates indicating a moderate genetic contribution. These findings emphasize the importance of integrating genetic and genomic approaches to enhance the bioactive delivery of blueberries and identify key genotypes and genetic factors. Biofortification of fruit crops can be done by using the different techniques for enhancing the targeted nutrients and vitamins are as given in the **Table 4**.

**Table 4:** Overview of Empowering Vital Fruit Crops with Enhanced Nutritional Contents.

Sl.No.	Fruit/Crop	Technique	Nutrient Focus	Reference
1.	Pomegranate	Breeding	Iron, Zinc, Vitamin C	Yadava et al., 2020
2.	Banana	Traditional Breeding, Genetic Engineering	Provitamin A, Iron, Zinc	Davey et al., 2009; Arango et al., 2010; Naqvi et al., 2009; Joshi et al., 2017a; Lobo et al., 2010; Shekhawat et al., 2012
3.	Mango	Breeding, Genetic Engineering, Agronomic Practices	Iron, Zinc, Calcium, Beta-Carotene	Padmesh et al., 2013; Saranya et al., 2017; Dhotra et al., 2021
4.	Guava	Traditional Breeding, Genetic Engineering	Vitamin C, Beta-Carotene, Iron, Zinc	Navarro-Tarazaga et al., 2016; Sathya et al., 2019
5.	Grape	Breeding, Genetic Engineering, Agronomic Practices	Polyphenols, Anthocyanins, Resveratrol, Vitamin C, K	Di Lorenzo et al., 2019; Vezzulli et al., 2007; Cortell and Kennedy, 2006
6.	Papaya	Genetic Engineering	Provitamin A, Iron, Zinc	Shankar et al., 2010; Maxwell et al., 2013
7.	Apple	Genetic Engineering, Nutrient Management	Antioxidants (Phenolics, Flavonoids, Anthocyanins)	Strand et al., 2018; Tagliavini et al., 2019; Tian et al., 2017; Flachowsky et al., 2010; Lobato-Gomez et al., 2021
8.	Pear	Breeding, Genetic Engineering, Agronomic Practices	Vitamins, Minerals, Antioxidants	Shankar et al., 2010; Cardoso et al., 2018; Liu et al., 2023; Zhang et al., 2021
9.	Aonla	Traditional Breeding, Genetic Engineering, Agronomic Practices	Vitamin C	Singh et al., 2022; Chaurasia et al., 2009; Iqbal et al., 2015
10.	Strawberry	Genetic Engineering, Agronomic Practices	Vitamin C, Sweetness	Singh et al., 2022; Borowski et al., 2016; Park et al., 2006; Kumar et al., 2012
11.	Citrus	Breeding, Genetic Engineering, Agronomic Practices	Vitamin C, Iron, Pro-Vitamin A	Salonia et al., 2020; Zhang et al., 2019; Pons et al., 2018, Pons et al, 2018; Bakshi et al., 2017; Devi et al., 2018
12.	Watermelon	Breeding, Genetic Engineering	Lycopene, Protein, Amino Acids	Perkins et al., 2001; Deng et al., 2023; Joshi et al., 2021
13.	Blueberries	Genetic Engineering, Environmental Factors	Phenolic Compounds, Flavonoids	Mengist et al., 2020

## 4 SWOT analysis for biofortification in fruit crops

Biofortification is a vital strategy for enhancing the nutritional value of fruits by increasing essential vitamins, minerals, and beneficial compounds. For instance, *Mangifera indica* (mango) has benefited from traditional breeding techniques that identify varieties rich in minerals like iron and zinc (Padmesh et al., 2013). Additionally, genetic engineering has successfully increased beta-carotene levels (Saranya et al., 2017), while agronomic practices such as micronutrient applications further enhance fruit quality (Dhotra et al., 2021). Similarly, *Psidium guajava* (guava) utilizes both traditional breeding for higher vitamin C and zinc content and genetic engineering to improve antioxidant levels, notably in the Paluma cultivar (Navarro-Tarazaga et al., 2016; Sathya et al., 2019).

Traditional breeding and genetic engineering also play significant roles in *Vitis vinifera* (grapes), where breeding increases antioxidants like polyphenols, and genetic modifications boost resveratrol levels (Di Lorenzo et al., 2019; Vezzulli et al., 2007). Agronomic practices contribute further to the nutritional content of these fruits (Cortell and Kennedy, 2006). In *Carica papaya* (papaya), genetic engineering aims to elevate provitamin A carotenoids and enhance disease resistance through targeted gene modifications (Shankar et al., 2010; Devit et al., 2010). Likewise, *Malus domestica* (apple) benefits from both traditional breeding and genetic engineering, with Arctic^®^ varieties offering resistance to browning and enhanced antioxidant levels (Strand et al., 2018; Lobato-Gomez et al., 2021).

*Pyrus communis* (pear) demonstrates a similar integration of traditional breeding and genetic modifications aimed at boosting vitamins and antioxidants, complemented by agronomic practices such as foliar calcium and zinc applications (Han et al., 2016; Cardoso et al., 2018; Liu et al., 2023). Meanwhile, *Phyllanthus emblica* (aonla) relies primarily on traditional breeding and agronomic practices for quality improvements, facing regulatory challenges in genetic engineering (Singh et al., 2022; Iqbal et al., 2015).

Advancements in *Fragaria × ananassa* (strawberry) include improvements in vitamin C content and sweetness achieved through genetic modifications and agronomic techniques like cycocel spraying (Borowski et al., 2016; Kumar et al., 2012). Citrus species (*Citrus* spp.) have similarly benefited from breeding and genetic engineering to enhance vitamin C and iron content, further improved by agronomic practices (Salonia et al., 2020; Zhang et al., 2019). For *Citrullus lanatus* (watermelon), breeding and genetic modifications have increased lycopene content, while emerging techniques such as nanoscale particles for nutrient enhancement show promise (Perkins et al., 2001; Deng et al., 2023). Lastly, *Vaccinium corymbosum* (blueberries) highlights the variability in phenolic compound bioaccessibility, influenced by genetic and environmental factors, underscoring the necessity for integrated approaches (Mengist et al., 2020).

These examples collectively illustrate the potential of combining traditional, genetic, and agronomic methods to comprehensively improve fruit nutrition. Continued research is crucial for optimizing these approaches to meet global nutritional needs effectively.

## 5 Challenges for biofortification of fruit crops

### 5.1 Micronutrient malnutrition associated health problems

Micronutrient malnutrition affects over 2 billion people globally, with deficiencies in vitamins and minerals like iodine, iron, zinc, and vitamin A posing significant health risks. Unlike macronutrients, which are needed in large amounts, micronutrients are essential in smaller quantities for proper bodily functions and development. Poor intake and absorption, exacerbated by factors such as inadequate food security and health services, lead to widespread deficiencies, particularly among pregnant women and young children. With the world population projected to reach 8 billion by 2030, addressing micronutrient malnutrition is crucial for improving public health, especially in developing countries.

A. Iron: Iron is essential for various cell functions, notably oxygen transport in hemoglobin. It is the leading cause of anemia, particularly affecting young women and children, with over 2 billion people impacted worldwide (Benoist et al., 2008). Symptoms of iron deficiency include fatigue and metabolic disruptions, while severe cases can lead to significant health complications.

B. Zinc: Zinc is a vital mineral required for cell division, growth, and immune function, yet it cannot be stored in the body (Frassinetti et al., 2006). Approximately 1.1 billion people are affected by zinc deficiency due to inadequate dietary intake. This deficiency is linked to various health issues, including impaired immunity, delayed wound healing, and emotional disturbances (Evans, 1986).

C. Iodine: Iodine is crucial for the production of thyroid hormones, which regulate metabolism. More than 2 billion people globally suffer from iodine deficiency, leading to conditions like goiter and developmental impairments in children (Delange, 1994). Insufficient iodine during pregnancy can result in neuro developmental issues for the offspring.

D. Vitamin A: Vitamin A is important for immune health, vision, and cell growth. Its deficiency affects 100–400 million preschool-aged children globally, resulting in significant health consequences, including blindness. Among pregnant women, vitamin A deficiency can led to night blindness and increased maternal mortality. Many developing countries rely on plant foods to meet their vitamin A needs.

E. Vitamin B: Vitamin B comprises eight forms, including B1, B2, B3, B5, B6, B8, B9, and B12, each acting as a co-factor in essential metabolic processes like carbohydrate metabolism and protein synthesis. Vitamin B6, in particular, is vital for protein metabolism, immune function, and neurotransmitter synthesis, but humans cannot produce it and must obtain it from dietary sources (Bryan et al., 2002). The rising prevalence of B6 deficiencies can lead to symptoms such as skin inflammation, fatigue, and depression.

F. Vitamin C: Vitamin C is a water-soluble vitamin predominantly sourced from plants, well-known for its immune-boosting properties and antioxidant effects. It acts as a co-factor in synthesizing collagen, cholesterol, and amino acids, and is also involved in energy metabolism (Perez-Massot et al., 2013; Maggini et al., 2017). Deficiency in vitamin C can lead to joint pain, connective tissue disorders, poor wound healing, and weakened immune response (Maggini et al., 2017).

G. Vitamin E: Vitamin E is a fat-soluble vitamin found in oil-rich foods like peanuts and sunflower seeds, and it can be stored in the body’s fat reserves. It serves as an antioxidant, regulates membrane lipid packaging, and is important for preventing diseases like cancer and cardiovascular conditions (Fitzpatrick et al., 2012). Deficiency typically occurs in individuals with fat metabolism disorders and can result in muscle weakness, hemolytic anemia, and neurological issues.

### 5.2 Factors affecting micronutrient availability

A. Soil and Plant Factors: Micronutrients travel from the soil to plants and then to humans, so the quality of the soil is very important. Key factors include soil texture, pH level, organic matter, and moisture. How well plants absorb nutrients depends on their root structure and connections with fungi (mycorrhizae). Some plants adapt to low nutrient levels by growing more roots or releasing acids to make nutrients more available (Zhang et al., 2010; Marschner, 2012).

B. Diet-Related Factors: When we eat plant foods, various factors affect how well our bodies absorb nutrients. These include the form of the nutrient, the type of food, and how different nutrients interact with each other. For example, vitamin C can help with iron absorption, while compounds like phytic acid can block it (Gibson, 2007). Individual factors like age, health, and genetics also play a role. If someone is low in iron or zinc, their body may absorb these nutrients better during that time (Hallberg and Hulthen, 2000). Malnutrition and infections can further hinder nutrient absorption (Johnson et al., 2008).

C. Food Fortification: To fight micronutrient malnutrition, adding essential nutrients to everyday foods—called food fortification—is a useful strategy (Allen et al., 2006). This method has been widely used in richer countries and is becoming more common in developing nations. For instance, Pakistan has started programs to add vitamins and minerals to wheat flour and cooking oil (Awan et al., 2013). These initiatives aim to improve nutrition without requiring people to change their diets significantly.

D. Biofortification of fruit crops faces several limitations, including issues related to bioavailability and the presence of antinutrients, which can hinder the absorption of essential micronutrients. For instance, compounds like phytic acid can form complexes with minerals such as iron and zinc, reducing their solubility and absorption in the human body. Additionally, consumer acceptance of biofortified crops, particularly those developed through transgenic methods, is often low due to concerns regarding genetically modified organisms. Regulatory challenges further complicate the production and distribution of these crops, as numerous regulations govern transgenic crops, creating barriers to market entry. Moreover, agronomic biofortification relies on the regular application of mineral fertilizers, which can be costly and may not be feasible for all farmers, potentially leading to environmental concerns and toxicity from repeated applications. Thus, while biofortification holds promise for improving nutritional quality, these limitations must be addressed to enhance its effectiveness and accessibility (Ofori et al., 2022).

## 6 Scope and future prospective

To take the edge off Malnutrition, Biofortification is the most economical way to reinforce the nutritional security to undernourished people across India and world. The technology is not only confined to Agronomic fortification but also it has explored various conventional and biotechnological interventions leading to Biofortification in commercially important crops. Collaboration of interdisciplinary subject specialists along with their dedicated effort is needed. As compared to conventional breeding, biotechnological approaches to fortify crops are less time consuming but on the other hand lacks socio-economic and public acceptance as some of its advance approaches like transgenic breeding involves transfer of gene across genera or species and approval from genetic engineering approval committee (GEAC) in countries like India is also difficult. A Biofortification effort based on multiple coordinated efforts from interdisciplinary group is required for reducing the malnutrition in different populations thereby reducing the global hunger index and nutrition.

Van et al., 2017 found that biofortification through genetic engineering is generally well accepted by the majority of beneficiaries, with consumers willing to pay a premium for biofortified crops, which is crucial for the successful implementation of biofortification as a health policy intervention. They emphasized that this approach offers good value for money, particularly when multiple micronutrients are targeted in a single crop, thereby enhancing its economic viability and appeal to both producers and consumers. Furthermore, the authors highlighted the necessity of upgrading local supply chains to fully exploit the potential of biofortification and ensure effective delivery to consumers, especially in domestic markets where trade issues may be less relevant. Lastly, they underscored the importance of addressing ethical considerations related to biofortification, which is essential for informing policymakers and stakeholders about the implications of genetic engineering and ensuring the equitable distribution of biofortified crops.

In order to make use of Biofortified crops in seed chain, strengthening of formal and informal farming system is needed which most of the countries are using for reducing malnutrition. Intensive efforts have to be made by the policy makers like fixation of minimum support price (MSP) in public sector to support and promote Biofortified varieties. Considering these challenges the coordinated efforts by public sector, private sector and farmers will serve as a powerful tool for eradication of malnutrition all over the world.

## 7 Conclusion

Biofortification of fruits through traditional breeding, genetic engineering, and agronomic practices has substantially improved their nutritional content. Traditional breeding enhances fruit quality by selecting varieties with naturally higher nutrient levels, while genetic engineering enables precise modifications to boost specific vitamins and compounds. Agronomic practices further support these improvements by optimizing cultivation conditions. Combining these methods provides a comprehensive approach to addressing nutrient deficiencies and enhancing fruit quality. Continued research and development are essential to refine these strategies and ensure their widespread application, ultimately improving global fruit nutrition and health outcomes.

## 8 Organizations involved

Many organizations are working to improve the nutritional quality of crops through biofortification. These include both national and international groups focused on fighting malnutrition and enhancing food security. Some key organizations involved in these efforts are CGIAR, which conducts agricultural research to reduce poverty and improve food security; CIAT, which aims to boost agricultural productivity in tropical areas; and CIMMYT, which specializes in maize and wheat research. The Food and Agriculture Organization (FAO) works globally to eliminate hunger and improve nutrition, while Harvest Plus is a leader in biofortification, focusing on increasing nutrients in staple crops. Other important organizations include GAIN, which aims to improve nutrition for vulnerable populations, UNICEF, which focuses on children’s nutrition, and the World Health Organization (WHO), which addresses global health issues, including malnutrition.
